# Development of a Charge-Multiplication CMOS Image Sensor Based on Capacitive Trench for Low-Light-Level Imaging

**DOI:** 10.3390/s23239518

**Published:** 2023-11-30

**Authors:** Olivier Marcelot, Marjorie Morvan, Antoine Salih Alj, Stephane Demiguel, Cedric Virmontois, Anne Rouvie, Magali Estribeau, Vincent Goiffon

**Affiliations:** 1 ISAE-SUPAERO, Université de Toulouse, F-31055 Toulouse, France; 2 Thales Alenia Space, 5 All. Des Gabians, F-06400 Cannes, France; 3 CNES, 18 Av. Edouard Belin, F-31400 Toulouse, France

**Keywords:** image sensors, CMOS image sensors, Charge Coupled Device (CCD), CMOS, electron multiplication, EMCCD

## Abstract

This paper presents an electron multiplication charge coupled device (EMCCD) based on capacitive deep trench isolation (CDTI) and developed using complementary metal oxide semiconductor (CMOS) technology. The CDTI transfer register offers a charge transfer inefficiency lower than $10^{-4}$ and a low dark current o $0.11\;\mathrm{nA}/{\mathrm{cm}}^{2}$ at room temperature. In this work, the timing diagram is adapted to use this CDTI transfer register in an electron multiplication mode. The results highlight some limitations of this device in such an EM configuration: for instance, an unexpected increase in the dark current is observed. A design modification is then proposed to overcome these limitations and rely on the addition of an electrode on the top of the register. Thus, this new device preserves the good transfer performance of the register while adding an electron multiplication function. Technology computer-aided design (TCAD) simulations in 2D and 3D are performed with this new design and reveal a very promising structure.

## 1. Introduction

The latest advances in complementary metal oxide semiconductor (CMOS) technologies have led to their adoption in image sensors. Compared to Charge Coupled Devices (CCD), CMOS image sensors offer a higher level of integration with on-chip CMOS functions and lower power voltage [1]. However, high sensitivity for low-level imaging in CMOS image sensors are still tricky to achieve, especially with EMCCD devices. Various kinds of noise sources are present in CMOS image sensors such as temporal and spatial noise, dark current or read noise, as well as the degradation of the signal-to-noise ratio when a small amount of photo-generated carriers have to be detected, which is very difficult to avoid. One solution is to amplify the signal before its charge-to-voltage conversion in order to minimize the effect of the noise sources and especially the dark current. This can be achieved thanks to electron multiplication in EMCCD [2]. Although a new kind of high performance low-light-level image sensor has been developed thanks to the advent of the quanta image sensor [3], EMCCDs are still wanted for particular applications such as time delay integration for earth observations.

The EMCCD using impact ionization was proposed in the 1980s [4], and it is known for its good capability to record a low-light-level scene [5,6,7] thanks to the multiplication of the amount of charges in the CCD register with a good noise control. The latest advances in this topic have been achieved thanks to the use of a buried channel and short poly-gap distances. For instance, the following performances have been recently demonstrated: a gain of 3% per stage, a CTI of 0.01, a dark current of 2–3$\;\mathrm{nA}/{\mathrm{cm}}^{2}$, and a Full Well Charge (FWC) of $116\;{\mathrm{ke}}^{-}$ [8]. Although few other CMOS EMCCD have been developed on various technologies [9,10,11,12], none of them relate to the development of EMCCD with deep trench-based devices. Therefore, in this paper, we would like to propose an innovative structure based on Capacitive Deep Trench Isolation (CDTI) [13]. The goal is to demonstrate a well-controlled charge multiplication in such a device.

A trench CCD has been proposed in 1989 [14] where electrons are carried between two trenches, but the development seems to be canceled. Then, a CCD-on-CMOS trench structure based on CDTI has been developed in 2020 [15], and this device is used for this study. In this particular concept, CDTIs shape the electrostatic potential, leading to carrier displacement in a buried channel mode. This device presents attractive characteristics such as a Charge Transfer Inefficiency (CTI) lower than $10^{-4}$ [15], a FWC of $57.6\;{\mathrm{ke}}^{-}$, a dynamic range of 78dB [16], and a dark current of $0.11\;\mathrm{nA}/{\mathrm{cm}}^{2}$ at room temperature for a pixel size of 3×12μm${}^{2}$. Therefore, this register combines a low CTI, a high FWC, and a low dark level. For comparison, state-of-the-art characteristics of CCD-on-CMOS devices are a CTI of $1\times 10^{-5}$, a FWC of $30\;{\mathrm{ke}}^{-}$, and a dark current of $3.7\;\mathrm{nA}/{\mathrm{cm}}^{2}$ [17].

Therefore, the aim of this new development is to propose an EMCCD device based on an innovating architecture and with state-of-the-art performances concerning the dark current and the CTI. Advantages and drawbacks of this device for electron multiplication are studied experimentally and with the TCAD simulations. Furthermore, a new structure device is suggested and investigated.

## 2. CDTI Device Description

### 2.1. Experimental Setup

**Figure 1:**
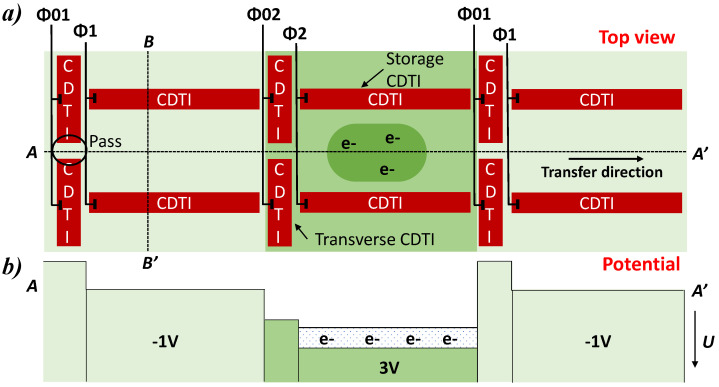
(**a**) Top schematic view of the CDTI phase register and (**b**) schematic representation of the electrostatic potential (U) profile along the transfer path. The schematics are redesigned from the reference [15].

The CDTI structure under investigation is a two-phase structure based on two kinds of CDTI: storage CDTIs along the path of the charge transfer and transverse CDTIs separated by a pass and perpendicular to the storage CDTIs (see Figure 1a). The transverse CDTI is used to create a lower potential barrier between phases thanks to the design. Thus, the charges can be stored in one phase even when adjacent phases are biased to the same voltage and transfer happens in one preferential direction as shown by the schematic representation of the potential in Figure 1b. Passivation of the Si-SiO2 interface defects of the CDTI edges is achieved by biasing the CDTI at $V_{CDTI}=-1\;V$ in inversion at low state, while the transfer is performed at $V_{CDTI}=3\;V$. In inversion, a thin hole layer accumulates at the Si-SiO2 interfaces, thus passivating the trench surfaces and leading to a very low dark current generation [18].

The n-well doping profile has a maximum doping concentration at about 1μm depth, which makes the charge storage possibly deep in the volume of the silicon, far from the surface. The n-well doping concentration has been adjusted in a way to allow for a full depletion of the buried layer, as described in the reference [15]. The n-well storage and transfer volume are delimited by a p+ pinning implantation on the top, as well as by the p-epitaxial layer at the bottom and laterally by the CDTI gates. These CDTI trenches are filled with p-doped polysilicon and contacted at their surface. CDTIs have a depth of 2.1μm and a width of 0.2μm.

**Figure 2:**
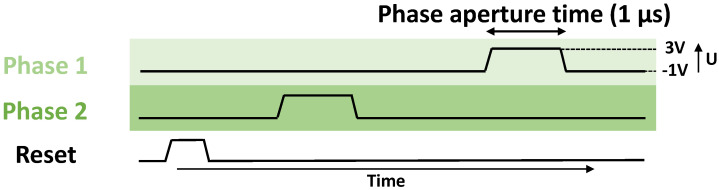
Two-phase timing diagram applied to the CDTI device with pulse duration of 1μs and rising/falling edge times of 10ns.

The transfer register is operated with the two-phase timing diagram presented in Figure 2. To transfer the integrated signal from phase 1 to phase 2, the last phase is biased to a positive voltage. The surface potential as well as the depletion potential between the CDTIs are lowered compared to the potential of the first phase. Then, the electrostatic potential gradient allows for the charge transfer from phase 1 to phase 2, similarly to classical CCDs.

**Figure 3:**
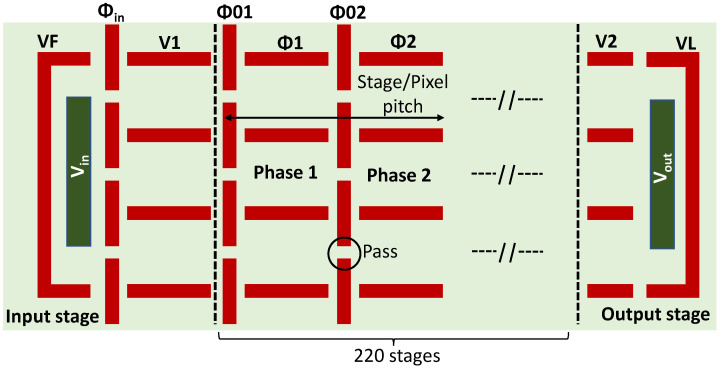
Top view schematic of the fabricated CDTI register layout. $V_{in}$ is the n+ injection node; $V_{out}$ is the n+ output node; VF and VL are CDTI surrounding $V_{in}$ and $V_{out}$, respectively; $\phi_{in}$ is a sampling barrier CDTI gate; and V1 and V2 are intermediate storage phases. The schematic is modified from [16].

The charge transfer device is based on three transfer fingers fabricated in STM IMG140 technology between the input stage and the output stage as presented in Figure 3. The three transfer fingers are used here to increase the charge transfer capacity. Several design variations in the two-phase shift register have been fabricated and address the pixel-stage pitch, the number of stages, and the pass width. The nominal design has a pitch of 12μm, 220 stages, and a pass width of 0.2μm. The input stage relies on an n+ drain injection node, followed by a sampling barrier CDTI gate $\phi_{in}$, allowing for the generation of synchronous charge pulses using a common diode cut-off technique [19]. The output stage is based on a DC-biased decoupling gate V2, followed by an n+ floating diffusion. The input and the output stages are surrounded by CDTI VF and VL to avoid parasitic collections. The floating diffusion is reset by means of a reset transistor and is read thanks to a source follower as can be found in the pixel arrays. The CDTI register is characterized at room temperature using a Cascade semi-automatic Prober equipped with a probe card and a Pulse Instrument data generator. The charge-to-voltage Conversion Factor (CVF) is evaluated with the common mean-variance method [20] at 22μV/e${}^{-}$. As this CVF does not allow us to reach the FWC because of the output stage saturation, a second lower CVF is implemented and is activated once the output voltage exceeds 600mV. This latter CVF allows for the full FWC evaluation. In order to quantify the correct number of electrons beyond 600mV, a corrective factor is applied. The CTI is evaluated by using the Extended Pixel Edge Response (EPER) method [20,21], which consists in measuring the deferred charges following an injected charge sequence. The dark current is measured by subtracting the reference level from the signal level without injection in operating conditions with a low and high state alternating as shown by the timing diagram.

### 2.2. Preliminary Measurements

**Figure 4:**
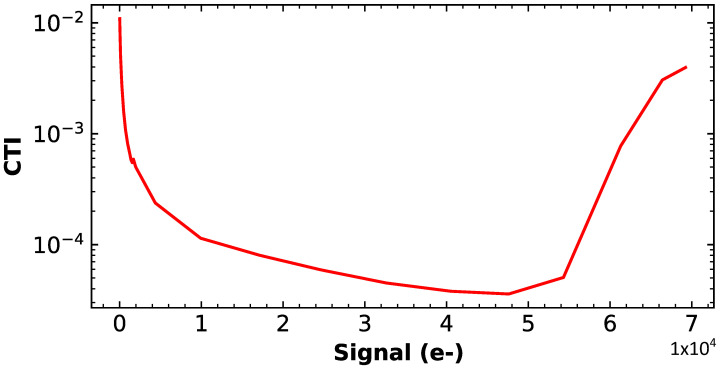
CTI measured by EPER against the injected charge with pulse duration of 1μs and rising/falling edges times of 10ns.

CTI and dark current measurements are performed to evaluate the performance of the CDTI register in an avalanche configuration, which was never performed before in any study. The CTI measurement is shown in Figure 4 and achieves low values under $1\times 10^{-4}$. A CTI increase is visible at low injection as it is usually the case in CCDs due to the presence of a trapped charge. At high injection, the CTI also increases and it is attributed to the change between the buried and surface transport regime. A dark current of $0.11\;\mathrm{nA}/{\mathrm{cm}}^{2}$ at room temperature is also measured, comparable to the best CCD-on-CMOS transfer registers. These measurements demonstrate the really good performance of the CDTI register operated in transfer mode.

## 3. EMCCD Operations

### 3.1. Experimental Setup

**Figure 5:**
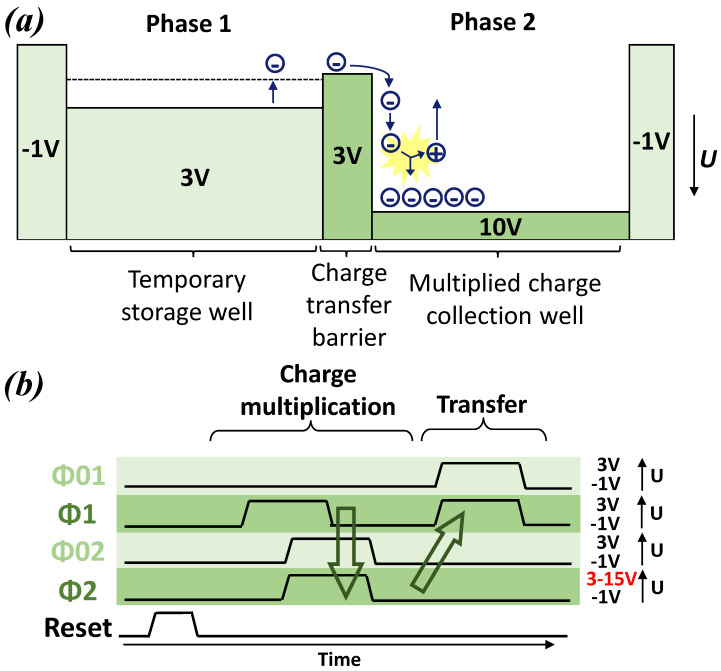
(**a**) Schematic representation of the electrostatic potential (U) profile along transfer path in avalanche mode and (**b**) timing diagram in avalanche mode for the CDTI register with low and high state potential (U), pulse duration of 2μs, and rising–falling edge times of 10ns.

In order to use the CDTI register under impact ionization conditions, some adjustments on the timing diagram need to be made. First, the electric field has to be strong enough to reach impact ionization and usually it needs to be greater than $1\times 10^{5}\;V/\mathrm{cm}$ [8,9,10]. Second, all charges to be multiplied have to go through a high and constant electric field during the transfer with the aim to reach a sufficient ionization rate [22]. The latter involves the creation of an intermediate storage well isolated from the phase and submitted to the high potential via a fixed potential barrier, as represented in Figure 5a. In our case, the storage and transverse CDTI can be operated independently, so phase 1 can be used as a temporary storage well and phase 2 can be as a multiplied charge collection well, while the transverse CDTI ϕ02 represents a charge transfer barrier. By doing so, the potential between ϕ02 and ϕ2 is kept constant during the avalanche, as requested. The timing diagram is adapted and is presented in Figure 5b. At the beginning of the multiplication phase, the charges are stored under phase 1 with the CDTIs ϕ1 biased at 3 V, then ϕ02 is biased at 3 V and ϕ2 is biased at a high voltage between 3 V and 15 V. The avalanche process begins when ϕ1 is biased at −1 V and the charges leave the temporary storage well to go through ϕ02. The impact ionization occurs between ϕ02 and ϕ2, where the high electric field region is located. Following this charge multiplication step, the charges are stored in an inversion condition in the volume between CDTI ϕ2 and can be normally transferred from phase 2 to phase 1.

### 3.2. Measurements

**Figure 6:**
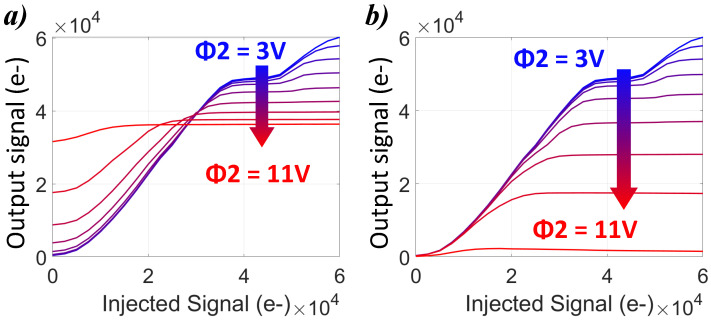
(**a**) Output signal measurement and (**b**) net electron signal as function of the injected signal for ϕ2 voltage between 3 V and 11 V after 220 stages.

The measurement of the output signal as a function of the injected signal in avalanche mode are presented in Figure 6a. Two aspects can be observed on this result. First, for a small injection level (charge<30% FWC), the output signal increases with the ϕ2 bias. On the contrary, for a strong injection level, the output signal decreases as the ϕ2 bias increases. The net signal is the subtraction between the output signal and the dark signal, and, as shown in Figure 6b, there is a reduction in this net transferred signal with the increase in ϕ2, where this phenomena is even more pronounced at a strong injection level. It suggests the absence of impact ionization of the injected charges.

In order to investigate the root cause of these results, TCAD simulations with Synopsys Sentaurus 2020.09-SP1 Software have been performed. To reduce the complexity and the simulation time, only one stage is simulated in 2D with doping profiles extracted from a Sprocess simulation with the same timing diagram and biases used for measurements. The injection is performed by controlling the bias of the n+ injection drain. To deplete the structure, the n+ output drain is biased at 3 V and one transfer cycle is simulated to empty the register. The device structure is simulated with the Structure Editor tool and electrical simulations are performed with Sdevice, with the following models activated [23]: hydrodynamic models for current densities, Philips unified model with doping dependence for the mobility, Band to band, Auger, SRH recombination with doping dependence, and an avalanche model for impact ionization.

**Figure 7:**
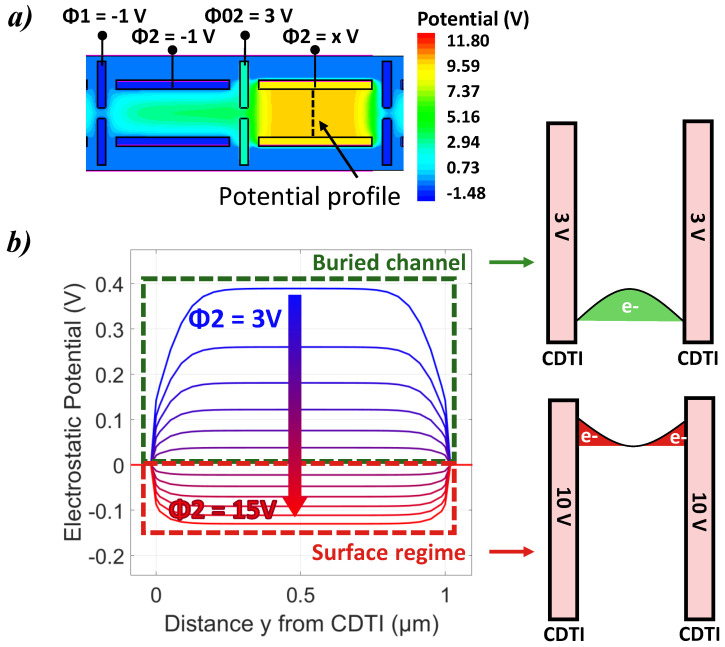
(**a**) Planar cross-section at 1μm depth of a 2D TCAD simulation of the electrostatic potential map. (**b**) Simulated potential profile between two CDTI as a function of ϕ2 for a given injection level, allowing us to see the buried channel and the surface regime.

In order to understand the decrease in the output signal for a large injection level, potential profiles between two storage CDTIs are extracted from the TCAD simulations at one injection level and depending on the ϕ2 bias. The results are displayed in Figure 7. Two different situations can be observed: a buried channel regime where the charges are confined between and away from the two CDTIs, and a surface regime where the charges are localized near the surfaces of both CDTI. The change from the buried channel to surface regime defines the FWC of the device, depending on ϕ2. Increasing the ϕ2 CDTI bias has the effect to shrink the potential well since the surface pinning dominates over the vertical p+/n/p pinning. Therefore, surface trapping is quickly promoted for the large bias on ϕ2.

**Figure 8:**
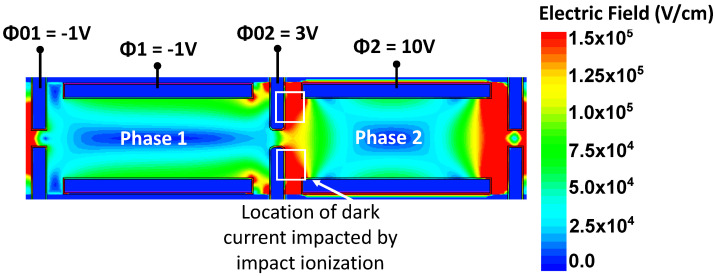
Planar cross-section at 1μm depth of the electric field map from a 2D TCAD simulation.

To investigate the increase in the output signal at a small injection, the TCAD simulations allow for the extraction of electrostatic potential and electric field maps during the avalanche process. As can be seen in Figure 8, the high electric field regions are located between CDTIs ϕ02 and ϕ2, close to the interfaces where the dark current is strongly suspected to be generated. An electric field of $2.10^{5}\;V/\mathrm{cm}$, high enough to create an impact ionization regime, is reached in the injected charge path (between CDTIs ϕ02) at ϕ2=16V, while a higher electric field is reached between ϕ02 and ϕ2, where the dark current generation is located. Therefore, as soon as ϕ2 is equal or higher than 6 V, impact ionization of the dark charge occurs, because of the bad localization of the high electric field, and a strong increase in the dark current is visible. Moreover, tests with different integration times have been performed and show a linear increase in the dark signal, with the integration time indicating the presence of a strong dark current. It can be noted that the strong electric field is also present at the top of the CDTI, where the p+ pinning layer is located. We can therefore reasonably think that this device is also affected by the clock-induced charge (CIC), with the latter being linked to the multiplication of holes under the effect of the strong electric field [24].

To conclude, two effects are observed here and hide the avalanche of the injected charges: the decrease in the FWC for large injections, and the increase in the dark current for a low injection level. In order to have a well-controlled multiplication, the Excess noise factor (ENF), i.e., the ratio between noise with and without the avalanche, must not exceed 1.4 [9]. In our case, for 220 stages, $60\;\mathrm{noise}\;e^{-}$ can be measured without the avalanche, so something like $84\;e^{-}$ should be measured with the activation of the avalanche. These values are largely exceeded. In the following, two solutions are tested with the aim to suppress these limitations.

### 3.3. FWC Optimization with Larger Pitches

**Figure 9:**
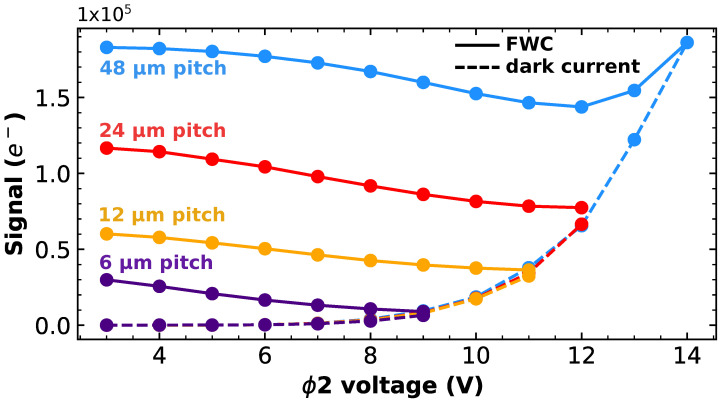
FWC signal and dark current signal after 220 stages as function of ϕ2 voltage for different stage pitches. Dark current curves are overlapping.

First, as the FWC is strongly reduced by the increase in the ϕ2 bias, registers with larger pitches are characterized with the goal to increase the FWC of the device. Three design variations are tested in addition to the nominal design: registers with 440 stages and with a 6μm pitch, 110 stages with a 24μm pitch, and 55 stages with a 48μm pitch. The results are presented in Figure 9 where the FWC signal and the dark current signal are plotted as a function of the ϕ2 bias for different stage pitches. As expected, the FWC increases when the stage pitch increases. Thus, for a large stage pitch, a higher ϕ2 bias can be used; however the dark current still limits the rise in the ϕ2 bias because it increases with an exponential behavior. Therefore, the larger pitch is not a solution to obtain the avalanche in those devices because of the strong dark current multiplication.

### 3.4. Dark Current Optimization with Lower Temperature

**Figure 10:**
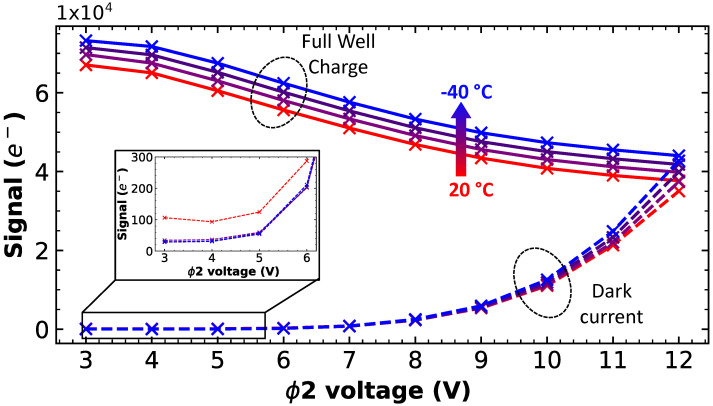
FWC signal and dark current signal as function of ϕ2 voltage for different temperatures (20 °C, 0 °C, −20 °C, and −40 °C). In inset, a zoom-in on the dark current signal as function of low ϕ2 voltage.

To prevent the dark current increase, measurements under a lower temperature are performed as presented in Figure 10. To do so, the devices are packaged in order to be usable in a thermal chamber, where the temperature is varied from 20 °C to −40 °C. A sensible effect on the FWC can be observed here: the FWC increases linearly as the temperature decreases. It can be attributed to the fact that electrons escape the potential well due to the thermionic emission; this phenomena increases with the temperature. Indeed, if the electron energy is high enough, it can jump into the Si-SiO2 interface region, lowering the FWC [20]. In addition, the freezing of the interface states may slow down the capture and emission process of traps, contributing to the observed tendency. Unfortunately, the dark current at a high ϕ2 voltage does not seem to be affected by the temperature reduction. The small shift for the high ϕ2 voltage is attributed to the FWC variation, as more dark-generated electrons can fill the larger FWC. For low ϕ2 biases, a reduction in the dark current can be observed from 20 °C to 0 °C, showing that temperature has an influence on the thermo-generated dark current, meaning that the increase observed for high ϕ2 biases is more likely due to the CIC. Consequently, it will not be possible to avoid a dark current multiplication in this device by simply lowering the temperature.

The solutions tested in this part were therefore not effective to achieve charge multiplication in these structures. For this reason, a design modification is proposed in the next section.

## 4. Modified CDTI Structure Investigated Using TCAD Simulations

The problem of the CDTI avalanche structures is that they offer large CDTI interface areas, which are not in the path of the injected charge and are exposed to the high electric field. This leads to an important dark current generation and CIC, which dominate the signal and prevent any charge multiplication observation. For a comparison purpose, 0.5μm${}^{2}$ of the silicon–oxide interface is exposed to the strong field for the planar electrodes’ design, as proposed by Dunford et al. [11], while 7.2μm${}^{2}$ are exposed to the high electric field in the CDTI registers. One also needs to notice that the oxide quality of CDTIs might be worse compared to surface gate oxides, leading to a higher dark current generation. With the aim to reduce the CDTI Si-SiO2 interface area exposed to the high electric field, solutions involving only CDTI components have been rejected because they do not allow for a strong electric field in the charge injection path and lead to large interface areas under the high electric field. Based on this conclusion, a new hybrid structure between the CDTI register and the classical CCD registers is suggested.

**Figure 11:**
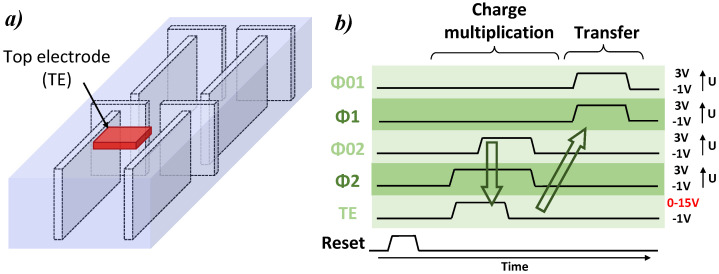
(**a**) Schematic of the new hybrid structure with the addition of the top electrode and (**b**) timing diagram associated with pulse duration of 2μs and rising/falling edges times of 10μns.

This structure is presented in Figure 11a. The modification consists in the addition of a top electrode (TE) above phase 2 biased at a high voltage to obtain the avalanche, while all other CDTI remains at normal biases. The CDTI transfer concept is kept and the device has the benefit of the very good transfer properties and low dark current in storage mode. The main advantage is the reduction in the surface exposed to a strong electric field to 0.12μm${}^{2}$ and also the use of surface gate oxide exposed to the high electric field instead of CDTIs, which is known to offer a better interface quality, and therefore, a lower dark current. In this structure, the strong electric field is at the same place as the multiplication of the charges, avoiding the problems of the higher dark charges’ multiplication. This hybrid structure involves two operation modes that can be seen on the timing diagram in Figure 11b: first, an avalanche mode, where the top electrode is biased at a high voltage and the charges are attracted near the TE surface, then, a classic *buried channel* charge transfer mode, when TE is not activated.

**Figure 12:**
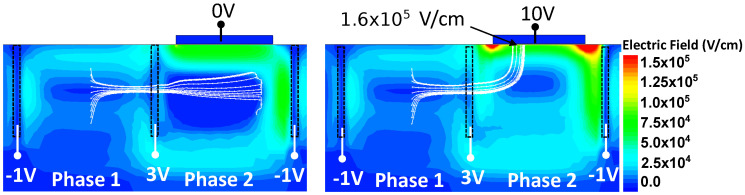
Cross-section view of the middle of one register in the transfer direction from a 3D TCAD simulation representing the electric field map with electron streamlines in white from the electrode of phase 1.

The new structure is studied thanks to the 3D TCAD simulations with the timing diagram presented in Figure 11b. CDTI are biased at −1 V for low state and 3 V for high state in the same way as previously for the charges’ transfer. The TE electrode is set at 0 V when disabled and is biased up to 15 V for the charge multiplication. Electric field cross-sections with the top electrode activated at 10 V or disabled at 0 V are presented in Figure 12. Streamlines are also shown and represent the path followed by electrons guided by the quasi-fermi gradient from a starting point which is the previous storage well. The streamlines show a correct charge transfer from one phase to the other one in transfer mode and from one phase to TE in avalanche mode. This observation is valid for $V_{TE}\geq 8\;V$. For a lower TE bias, the electrons remain partly trapped in potential pockets in the buried channel.

**Figure 13:**
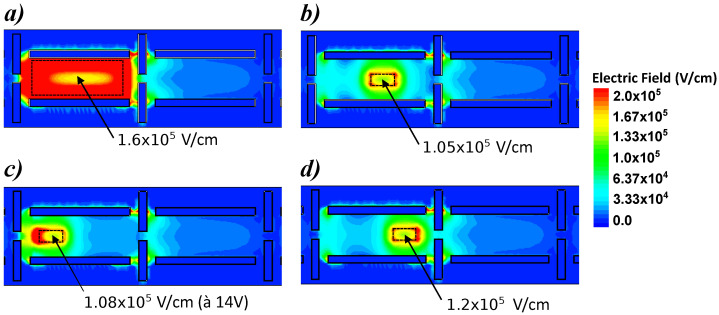
Planar cut at 0.05μm from the top of the register of the electric field TCAD simulation with different sizes of TE biased at 15 V.

As the structure is validated regarding charge transfer behavior, it can be studied more precisely for charge multiplication thanks to variations on TE positions and sizes as well as its optimum operating voltage. The optimization of TE size and position are performed with the simulations displayed in Figure 13. This figure shows planar cut at 0.05μm depth from the top of the register for a better understanding of electric field distributions. Comparison between large electrode (Figure 13a) of 2.4×0.9μm${}^{2}$ and small electrode of 0.6×0.2μm${}^{2}$ (Figure 13b) are represented as well as the small electrode at different positions under phase 2 (Figure 13c,d). In order to limit the charge multiplication of the dark current, the high electric field should be far from the CDTIs. At $V_{TE}=15\;V$, in the case of a large electrode, an electric field of $3.2\times 10^{5}\;V/\mathrm{cm}$ is reached on the edges of the CDTI whereas it is equal to $1.6\times 10^{5}\;V/\mathrm{cm}$ in the center. The injected charges coming mainly to the middle of the electrode, and the dark current coming from the interfaces of the CDTI, this structure will induce a higher dark charges’ multiplication compared to the injected charges. For the small electrode centered in the middle of the phase 2, TE is far enough to the CDTI edges to avoid this phenomenon and the multiplication of the dark current should be limited. Moreover, the electric field must be as homogeneous as possible under all the TE area in order to obtain a similar impact ionization under the TE center and near the edges. The goal is to avoid areas of the high electric field outside the localization of signal electrons. According to these simulations, an electric field variation between the center, and the edges of 33% with the small electrode and 50% with the large electrode is visible. Therefore, a small electrode at the center of the phase would be better to limit the dark current generation.

**Figure 14:**
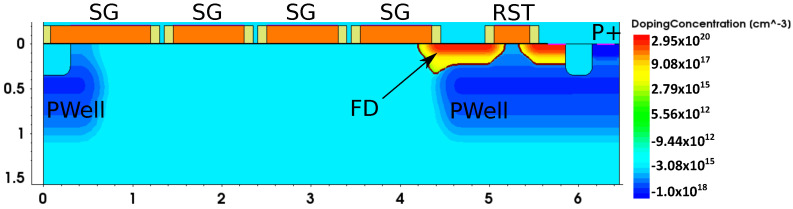
TCAD 2D distribution of doping concentration of the surface CCD device used for surface gate oxide calibration. SG is for storage gate, RST is for reset transistor, FD is the floating diffusion, and P+ is the ground contact.

In order to confirm it, additional 2D simulations are performed with the aim to extrapolate the dark current generation in avalanche mode. For this purpose, the TCAD simulation is calibrated according to the dark current measurements performed on the same technology:

- dark current on CDTI structure until $V_{\phi 2}=11\;V$. The test structure is identical to the one presented in Figure 8
- dark current on a surface gate structure until $V_{SG}=8\;V$. A cross-section view of the simulated and measured structure is given in Figure 14.

**Figure 15:**
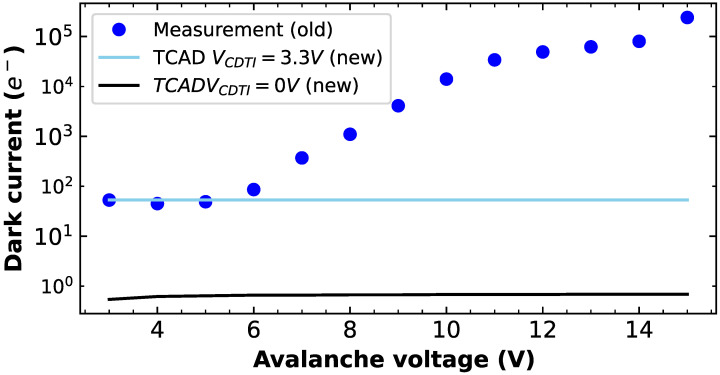
Comparison of dark current measurements on the CDTI structure and simulations on the new CDTI structure, including the TE gate for two CDTI biases. *Old* refers to the initial CDTI register and *new* refers to the new design with TE gate.

For these simulations, the surface SRH model is activated and the parameter $S_{0}$ controlling the surface recombination velocity [25] is calibrated at a low gate voltage, then traps are introduced if necessary at the Si-SiO2 interfaces with tunable concentrations in order to match with the measurements for all gate voltages. The 3D dark current value is deduced thanks to the multiplication by the third dimension of the simulated device. The calibration leads to the following parameters for the CDTI interface: $S_{0}=2000\;\mathrm{cm}/s$ and $\left[trap\right]=3.65\times 10^{12}\;/{\mathrm{cm}}^{2}$. For the surface gate structure, it is found that $S_{0}=20\;\mathrm{cm}/s$ and no traps are needed due to the better oxide quality. Then, taking into account these calibrated parameters and the geometry of the new structure with the smallest TE gate, it is possible to predict the dark current generation in avalanche mode. The results are shown in Figure 15 and demonstrate a much lower dark current with the new structure. Indeed, on one hand, the oxide area under the high electric field is reduced, and on the other hand, the oxide under the high electric field has a higher quality and the dark current is lower. The effect is even more pronounced if the storage CDTI ϕ2 is biased at 0 V during the avalanche process; however, this latter configuration should be demonstrated in the measurements.

**Figure 16:**
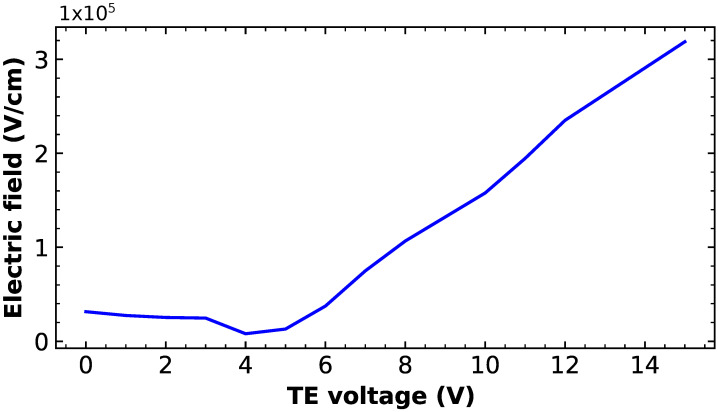
Electric field vs the TE voltage under TE at the depth where the quasi-fermi gradient is maximum for an oxide thickness of 18μm.

In order to address the minimum TE operating voltage needed to obtain the avalanche, the simulations of the electric field under the TE electrode as a function of the TE voltage are performed. For this purpose, the oxide thickness used under TE is 18 nm, with the goal to avoid any oxide breakdown. The electric field value is extracted at the depth where the quasi-fermi gradient is maximum. Therefore, the curve of the electric field as a function of the TE voltage is presented in Figure 16 and shows that the electric field under TE exceeds $1\times 10^{5}\;V/\mathrm{cm}$ for $V_{TE}\geq 8\;V$. From this voltage, the electrons are exposed to a sufficient electric field over a distance ranging from 30 to 100 nm; thus, the avalanche regime should be reached under these conditions. The expected impact ionization probability at $V_{TE}=12\;V$, inducing an electric field of $2.3\times 10^{5}\;V/\mathrm{cm}$ and taking into account the analytical models given by Moll and Overstraeten [26], is $1800\;{\mathrm{cm}}^{-1}$. Knowing that the register has 220 stages, it should lead to an expected multiplication gain of 50.

This newly developed structure looks very promising, as it combines advantages of the CDTI register and the impact ionization feature. However, measurements have to be carried out on that device in order to confirm the TCAD simulations and to evaluate the EMCCD key parameters. A test vehicle including this design is under realization and should be characterized in the next few months.

## 5. Conclusions

The CTI and the dark current have been measured on a CDTI transfer register, showing a CTI lower than $10^{-4}$ and a dark current of $0.11\;\mathrm{nA}/{\mathrm{cm}}^{2}$. This register has been tested with an adapted timing diagram in order to obtain an electron multiplication similar to the EMCCD devices. Several problems were highlighted, such as the decrease in the FWC and the strong dark current increase with the rise of CDTI gate voltages for reaching the avalanche state. Especially, the strong dark current generation is imputed to the large CDTI Si-SiO2 interfaces that cannot be reduced. These limitations do not allow for the observation of signal multiplication in these structures, so a modified structure has been proposed thanks to a hybrid design between classical CCD and CDTI. This new structure keeps the very good transfer and dark current property of the CDTI registers, and the TCAD simulations show a drastic reduction in the dark current thanks to smaller Si-SiO2 areas subjected to the high electric field and to the use of surface oxide instead of CDTI oxide. This structure is currently under realization and the first measurement should be performed in the next few months. This work demonstrates that the adaptation from a known 2D EMCCD device to a vertical and three-dimensional EM-CDTI device is not easy, although the CDTI device offers challenging performances.

## Data Availability

No new data were created or analyzed in this study. Data sharing is not applicable to this article.
